# Insomnia

**Published:** 1896-04

**Authors:** 


### Insomnia.

Insomnia is really a mere symptom, and will no more be treated *per se* by the intelligent practitioner than the eruption of an infectious fever or the diarrhœa of typhoid fever. The great duty of the medical man is to trace it to its causes and its associations, and to deal with these. If it follows influenza, it must be regarded, like all the other *sequelæ* of that protean disease, with some patience, but with much conviction that it will yield, sooner or later, to sound treatment. A very important point is to ascertain whether the insomnia is attended with pyrexia or otherwise, for of all means for producing restlessness, and marring the night's repose an increase of two or three degrees in the temperature is among the most effective. Apart from general pyrexia, it is well to note all local peculiarities of heat, whether in the direction of excess or defect—cold feet, a hot bed, etc.—and to deal with them accordingly. It is, of course, equally important to ascertain any error of function that can reasonably be associated with such a symptom. Such errors may frequently be found in the gastric or renal or hepatic functions, and their removal will quickly alter the whole complexion of the patient's life both by night and by day.—*Lancet.*

